# Assessment of the scalability of a microtiter plate system for screening of oleaginous microorganisms

**DOI:** 10.1007/s00253-018-8920-x

**Published:** 2018-04-11

**Authors:** Gergely Kosa, Kiira S. Vuoristo, Svein Jarle Horn, Boris Zimmermann, Nils Kristian Afseth, Achim Kohler, Volha Shapaval

**Affiliations:** 1 Faculty of Science and Technology, Norwegian University of Life Sciences, Postbox 5003, 1432 Ås, Norway; 2 Faculty of Chemistry, Biotechnology and Food Science, Norwegian University of Life Sciences, Postbox 5003, 1432 Ås, Norway; 3 Nofima AS, Osloveien 1, 1433 Ås, Norway

**Keywords:** Duetz-microtiter plate system, High-throughput screening, Oleaginous microorganism, Scalability, Bioreactors

## Abstract

Recent developments in molecular biology and metabolic engineering have resulted in a large increase in the number of strains that need to be tested, positioning high-throughput screening of microorganisms as an important step in bioprocess development. Scalability is crucial for performing reliable screening of microorganisms. Most of the scalability studies from microplate screening systems to controlled stirred-tank bioreactors have been performed so far with unicellular microorganisms. We have compared cultivation of industrially relevant oleaginous filamentous fungi and microalga in a Duetz-microtiter plate system to benchtop and pre-pilot bioreactors. Maximal glucose consumption rate, biomass concentration, lipid content of the biomass, biomass, and lipid yield values showed good scalability for *Mucor circinelloides* (less than 20% differences) and *Mortierella alpina* (less than 30% differences) filamentous fungi. Maximal glucose consumption and biomass production rates were identical for *Crypthecodinium cohnii* in microtiter plate and benchtop bioreactor. Most likely due to shear stress sensitivity of this microalga in stirred bioreactor, biomass concentration and lipid content of biomass were significantly higher in the microtiter plate system than in the benchtop bioreactor. Still, fermentation results obtained in the Duetz-microtiter plate system for *Crypthecodinium cohnii* are encouraging compared to what has been reported in literature. Good reproducibility (coefficient of variation less than 15% for biomass growth, glucose consumption, lipid content, and pH) were achieved in the Duetz-microtiter plate system for *Mucor circinelloides* and *Crypthecodinium cohnii*. *Mortierella alpina* cultivation reproducibility might be improved with inoculation optimization. In conclusion, we have presented suitability of the Duetz-microtiter plate system for the reproducible, scalable, and cost-efficient high-throughput screening of oleaginous microorganisms.

**Electronic supplementary material:**

The online version of this article (10.1007/s00253-018-8920-x) contains supplementary material, which is available to authorized users.

## Introduction

High-throughput screening (HTS) of microorganisms and cell cultures is an important step in the development of sustainable bioprocesses. Shake flasks have been the standard for screening of microbes, substrates, and growth conditions for a long time. Due to advances in metabolic engineering, the number of strains to be tested have increased significantly, making the throughput capacity of the shake flask cultures insufficient (Knudsen 2015; Silk et al. 2010). Recent developments in the miniaturization of fermentation systems have opened new opportunities in HTS, saving time and cost for bioprocess—and product development (Lübbehüsen et al. 2003). Microtiter plate-based systems (MTPS), with either 24-, 48-, or 96-well plates, are the most commonly used initial screening platform in biotechnology due to their simplicity, high throughput, good reproducibility, and automation possibilities (Long et al. 2014; Sohoni et al. 2012; Wu and Zhou 2014) It has been reported that the variability of extracellular metabolite production by filamentous microorganisms in MTPS is significantly lower than that in shake flasks (Linde et al. 2014; Siebenberg et al. 2010; Sohoni et al. 2012). Commercial HTS microtiter plate systems differ by monitoring and control options of process parameters (pH, DO, feeding, metabolites), throughput, instrument, and running cost (Long et al. 2014). Sophisticated, state-of-the-art MTPS with built-in optical sensors aim to mimic bioreactor cultivation environment. Good scalability has been reported in these systems up to 15 m^3^ bioreactors; however, most of these studies have been performed with unicellular microorganisms (bacteria and yeasts) (Back et al. 2016; Kensy et al. 2009; Knudsen 2015; Long et al. 2014; Lübbehüsen et al. 2003; Posch et al. 2013; Silk et al. 2010). Scalability of filamentous fungi from MTPS to bioreactors is rarely discussed and the few studies performed to date were performed at very a low substrate concentration (i.e., 5 g/L glucose) (Knudsen 2015). Application of optical online sensors in MTPS for the screening of filamentous fungi is problematic due to adherent wall growth and complex growth morphology. For these reasons, at/off-line bioprocess monitoring of filamentous fungi in MTPS is a more viable approach (Posch et al. 2013).

Duetz-MTPS is a simple and low-cost HTS system that consists of standard microplates (24, 48, or 96 wells) combined with a plate cover that enables sufficient gas transfer and prohibit extensive evaporation and cross-contamination of strains during cultivations (Duetz et al. 2000). The system offers very high throughput since MTPs can be stacked in a shaker. However, the system is considered less scalable due to a lack of control options and is therefore mainly used for initial strain selection based on end-point productivities (Long et al. 2014; Sohoni et al. 2012). In a recent study, we have evaluated the cultivation of *Mucor circinelloides*, *Umbelopsis isabellina*, and *Penicillium glabrum* oleaginous filamentous fungi in the Duetz-MTPS, resulting in good reproducibility and kinetics (Kosa et al. 2017).

The aim of the current study is to compare growth and lipid production of oleaginous microorganisms in Duetz-MTPS to controlled stirred-tank bioreactors. For this purpose, we selected the following oleaginous microorganisms: filamentous fungi *Mucor circinelloides* and *Mortierella alpina*, and heterotrophic microalga (marine dinoflagellate) *Crypthecodinium cohnii.* The selected microorganisms are producers of high-value polyunsaturated fatty acids (PUFAs), such as gamma-linolenic acid (GLA, C18:3n6), arachidonic acid (ARA, C20:4n6), and docosahexaenoic acid (DHA, C22:6n3) and have been used worldwide in nutraceutical products (Ratledge 2013). According to our knowledge, this is the first comparison of oleaginous microorganisms grown in Duetz-MTPS and in controlled stirred-tank bioreactors. We also show how Fourier transform infrared (FTIR) spectroscopy can be used in combination with the Duetz-MTPS for high-throughput characterization of oleaginous filamentous fungi and microalgae.

## Materials and methods

### Microorganisms

*Mucor circinelloides* VI 04473 was obtained from the Norwegian School of Veterinary Science (Oslo, Norway), while *Mortierella alpina* ATCC 32222 and *Crypthecodinium cohnii* ATCC 40750 were obtained from the American Type Culture Collection (Manassas, USA).

### Media and growth conditions

For inoculum preparation*,* filamentous fungi *M. circinelloides* and *M. alpina* were first cultivated on malt extract and potato dextrose agar, while dinoflagellate *C. cohnii* was maintained statically on an ATCC 2076 medium consisting of 4 g/L yeast extract (YE, Oxoid, Hampshire, England), 12 g/L glucose, and 25 g/L sea salts (Sigma-Aldrich, St Louis, USA). All cultures were incubated at 25 °C for 7 days. The inoculum medium for bioreactor experiments contained 40 g/L glucose—10 g/L YE for *M. circinelloides*, 20 g/L glucose—10 g/L YE for *M. alpina*, and ATCC 2076 medium for *C. cohnii*. 0.5- and 2-L shake flasks (baffled for fungi) were filled in with 150 and 625 mL inoculum media, respectively. Flasks were inoculated with fungal spores from Petri dishes or with 10 *v*/*v*% 7 days old *C. cohnii* seed culture and were grown at 25 °C for 2–4 days at shaking speed 100–150 rpm.

The lipid production media for *M. circinelloides* contained 80 g/L glucose and 3 g/L YE, for *M. alpina* it contained 60 g/L glucose and 10 g/L YE, while for *C. cohnii*, it consisted of 60 g/L glucose, 5 g/L YE, and 25 g/L sea salts. Fungal lipid production media also contained (g/L): KH_2_PO_4_ 7, Na_2_HPO_4_ 2, MgSO_4_.7H_2_O 1.5, CaCl_2_.2H_2_O 0.1, (from 1000× concentrated stock solution): FeCl_3_.6H_2_O 0.008, ZnSO_4_.7H_2_O 0.001, CoSO_4_.7H_2_O 0.0001, CuSO_4_.5H_2_O 0.0001, MnSO_4_.5H_2_O 0.0001 (Kavadia et al. 2001). Chemicals except YE were bought from Merck (Darmstadt, Germany). In case of *M. alpina*, 3.5 g/L KH_2_PO_4_ and 1 g/L Na_2_HPO_4_ were used. The chemical composition of lipid production media was the same for all tested cultivation scales (Duetz-MTPS—2.5 mL, benchtop fermenter—1.5 L, and pilot scale fermenter—25 L). Demineralized water was used for media preparation in Duetz-MTPS and benchtop bioreactor, while tap water was used in the pre-pilot scale bioreactor. pH of production media after autoclaving were 6.6, 6.1, and 6.3 for *C. cohnii*, *M. circinelloides*, and *M. alpina* respectively and pH was only controlled in bioreactors. The evolution of pH in Duetz-MTPS cultivations can be found in Fig. S4.

Cultivations in Duetz-MTPS were performed in autoclaved 24-square polypropylene deep well MTPs (total volume per well 11 mL) with low evaporation version sandwich cover (Enzyscreen, Heemstede, Netherlands). All wells were filled in with sterile lipid production medium and were incubated with 10–250 μL fungal spore or microalga suspensions, resulting in 2.5 mL final volume. The final concentrations were 5·10^8^ and 5·10^7^ of spores/mL for *M. circinelloides* and *M. alpina*, respectively. For *C. cohnii*, the final concentration was 5·10^6^ cells/mL. MTPS were incubated at 28 °C at 300 rpm (circular orbit 0.75″ or 19 mm) in an Innova 40R refrigerated desktop shaker (Eppendorf, Hamburg, Germany) for 7–8 days, and each day, one plate was removed for the analysis of biomass and supernatant. To compare performance with bioreactor runs, the merged content of the wells was used, while reproducibility in MTPS were tested by measuring three individual wells.

The bioreactor cultivations were performed in 2.5 L total volume glass fermenter (Minifors, Infors, Bottmingen, Switzerland) and 42 L total volume stainless steel in situ sterilizable fermenter (Techfors-S, Infors) with working volumes of 1.5 and 25 L, respectively (working volumes are used for referring to benchtop and pre-pilot scale fermentations in the following). Autoclaved and in situ sterilized media were inoculated with 10 and 4 *v*/*v*% (in benchtop and pre-pilot bioreactors, respectively) of the abovementioned shake flask inoculums. Glucose and trace element solutions were sterilized separately from the YE-salts solution and combined afterwards (same procedure in Duetz-MTPS).

For mixing, the benchtop and pilot fermenter were equipped with two and three 6-blade Rushton turbines, respectively. Temperature for all cultivations was 28 °C. pH was monitored with a pH probe (Mettler-Toledo, Greifensee, Switzerland) and was kept at 6.0 for *M. circinelloides*, *M. alpina* and 6.5 for *C. cohnii* with the automatic addition of 1 M NaOH and 1 M H_2_SO_4_ (for fungi) or 1 M HCl (for microalga). Dissolved oxygen (DO) was monitored with Hamilton (Bonaduz, Switzerland) and Mettler-Toledo polarographic oxygen sensors (in 1.5 L and 25 L bioreactors) and was maintained above 20% of the saturation with the automatic control of stirrer speed (300–600 rpm or 100–600 rpm for microalga). Off-gas analysis was performed with a FerMac 368 (Electrolab Biotech, Tewkesbury, UK) and Infors gas analyzers connected to the off-gas condenser of the glass and stainless steel fermenters, respectively. Cultures were aerated through a sparger at 0.5 VVM for fungi (0.75 and 12.5 L/min) and 1.0 VVM (1.5 L/min) for the microalga. Foam was controlled via a foam sensor with five times diluted Glanapon DB 870 antifoam (Busetti, Vienna, Austria).

*M. alpina* and *C. cohnii* had two parallel runs in the glass fermenters, while in case of *M. circinelloides*, only a single run was performed in a 1.5 L bioreactor due to technical problems. The cultivation of microalga *C. cohnii* was performed in MTPS and glass bioreactors, but not in the pre-pilot bioreactor due to the corrosive nature of ATCC 2076 medium for stainless steel (Behrens et al. 2010; Hillig et al. 2013).

### Microscopy

Micrographs were recorded with a DM6000B microscope (Leica Microsystems, Wetzlar, Germany) in bright-field and fluorescence mode after Nile-red staining according to the previously described protocol (Kosa et al. 2017).

### Optical density measurement

Optical density (OD) of *C. cohnii* was measured (after proper dilution) at 600 nm with a SPECTROstar Nano UV/Vis microplate reader (BMG Labtech, Ortenberg, Germany). A calibration of OD versus cell dry weight (g/L) was performed (Figure S3).

### Preparation of fungal biomass for FTIR analysis and lipid extraction for GC fatty acid analysis

Fungal biomass from MTPS and 1.5 L cultivations were filtered through a Whatman No. I filter paper in a vacuum setup (GE Whatman, Maidstone, UK), while in case of the 25 L cultivations, a 75-μm aperture test sieve was used (Endecotts, London, UK) for biomass separation. After filtration, the fungal biomass was washed thoroughly with distilled water. In case of microalga *C. cohnii*, the biomass was separated from the medium by centrifugation at 3000 rpm and it was washed once with distilled water. In the next step, the fungal and algal biomass was frozen at − 20 °C and then was lyophilized overnight in an Alpha 1-2 LDPlus freeze-dryer (Martin Christ, Osterode am Harz, Germany) at − 55 °C and 0.01 mbar pressure. The freeze-dried biomass was also used to calculate cell dry weight (CDW, g/L). Approximately 10 mg of freeze-dried biomass was transferred into 2-mL screw-cap tubes containing 500 μL distilled water and 250 ± 30 mg acid-washed glass beads (800 μm, OPS Diagnostics, Lebanon, USA). Biomass was then homogenized for 1–2 min in a FastPrep-24 high-speed benchtop homogenizer (MP Biomedicals, USA) at 6.5 m s^−1^. This homogenized fungal suspension was used for HTS-FTIR analysis. Lipid extraction protocol was performed according to previously described protocol (Kosa et al. 2017).

### Fourier transform infrared spectroscopy

FTIR analysis of freeze-dried and homogenized fungal biomass was performed with the High-Throughput Screening eXTension (HTS-XT) unit coupled to the Vertex 70 FTIR spectrometer (both Bruker Optik, Germany) in transmission mode (Kosa et al. 2017). Technical replicate spectra were averaged and then EMSC corrected (Kohler et al. 2005). For peak height determination second derivative (Savitzky-Golay, 2nd degree polynomial, 9 windows size) and EMSC correction were applied (Zimmermann and Kohler 2013). All pre-processing methods were performed using The Unscrambler X 10.5 (CAMO Software, Oslo, Norway).

### GC-FID fatty acid analysis

Determination of lipid content of fungal biomass (FAME content) and fatty acid composition analysis were performed with a HP 6890 gas chromatograph (Hewlett Packard, Palo Alto, USA) equipped with an SGE BPX70, 60.0 m × 250 μm × 0.25 μm column (SGE Analytical Science, Ringwood, Australia) and flame ionization detector (FID) (Kosa et al. 2017). For identification and quantification of fatty acids, the C4-C24 FAME mixture (Supelco, St. Louis, USA), C13:0 tridecanoic acid (Sigma-Aldrich), and C23:0 tricosanoic acid (Larodan, Solna, Sweden) internal standards were used.

### HPLC analysis

Glucose in the fermentation supernatant was quantified by using an UltiMate 3000 UHPLC system (Thermo Scientific, Waltham, USA) equipped with RFQ-Fast Acid H + 8% (100 × 7.8 mm) column (Phenomenex, Torrance, USA) and coupled to a refractive index (RI) detector. Samples were filter sterilized and subsequently eluted using isocratic method at 1.0 mL/min flow rate in 6 min with 5 mM H_2_SO_4_ mobile phase at 85 °C column temperature.

### Data analysis

Biochemical similarities between biomass samples were estimated using principal component analysis (PCA) of either GC or FTIR data. PCA data analysis was performed using The Unscrambler X 10.5 (CAMO Software, Oslo, Norway).

## Results

Scalability of the cultivation of the three oleaginous model organisms, *C. cohnii*, *M. circinelloides*, and *M. alpina* from Duetz-MTPS to controlled stirred-tank bioreactors were evaluated based on the following characteristics: (a) cell morphology, (b) growth and substrate consumption rates, (c) biomass concentration and lipid content of biomass, and (d) fatty acid composition. The biochemical composition of cells were also measured by FTIR spectroscopy. In addition to scalability, the reproducibility of cultivations in Duetz-MTPS was investigated.

## Morphology

**Fig. 1:**
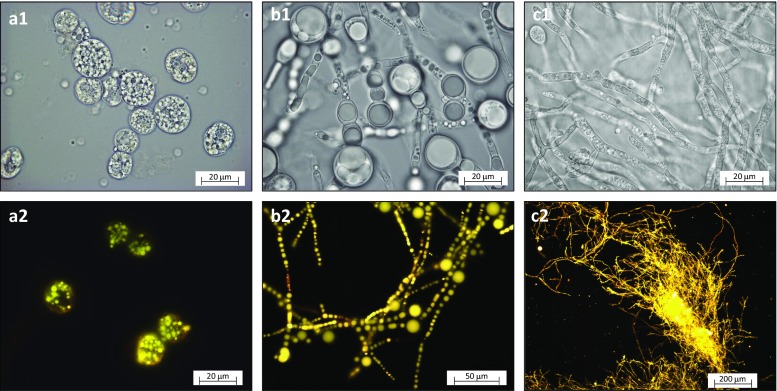
Morphology of oleaginous microalga and fungi. (a1, a2) *C. cohnii* (Duetz-MTPS, 120 h). (b1, b2) *M. circinelloides* (Duetz-MTPS, 168 h). (c1, c2) *M. alpina* (25 L bioreactor, 145 h) in bright-field (1) and fluorescence mode after Nile-red staining (2)

The morphology of *C. cohnii* was similar in the MTPS and in the 1.5 L bioreactor: a combination of motile cells with two flagella and bigger static cells (Mendes et al. 2009) (Figure S1 a-b). The cells had a high number of oval starch granules (Deschamps et al. 2008), and towards the end of the fermentation circular lipid bodies (Fig. 1(a1, a2)). Micrographs of *C. cohnii* show that algal cells are sensitive to shear stress, resulting in cell bursting (Figure S1 c). *M. circinelloides* is a dimorphic fungus capable of growing both in filaments and yeast-like single cells, depending on environmental conditions (Lübbehüsen et al. 2003). In stirred bioreactors, the single cell form was more pronounced than in MTPS, probably caused by higher shear forces in the bioreactors (Figure S2). The predominant filamentous form looked similar at all tested scales, with different size (up to 15 μm) of lipid bodies in the hyphae (Fig. 1(b1, b2)). *M. alpina* also had similar morphology at all tested scales: fluffy pellets with a high number of small lipid bodies in the hyphae (max diameter 3 μm) (Fig. 1(c1, c2)).

## Glucose consumption and biomass production rates

**Fig. 2:**
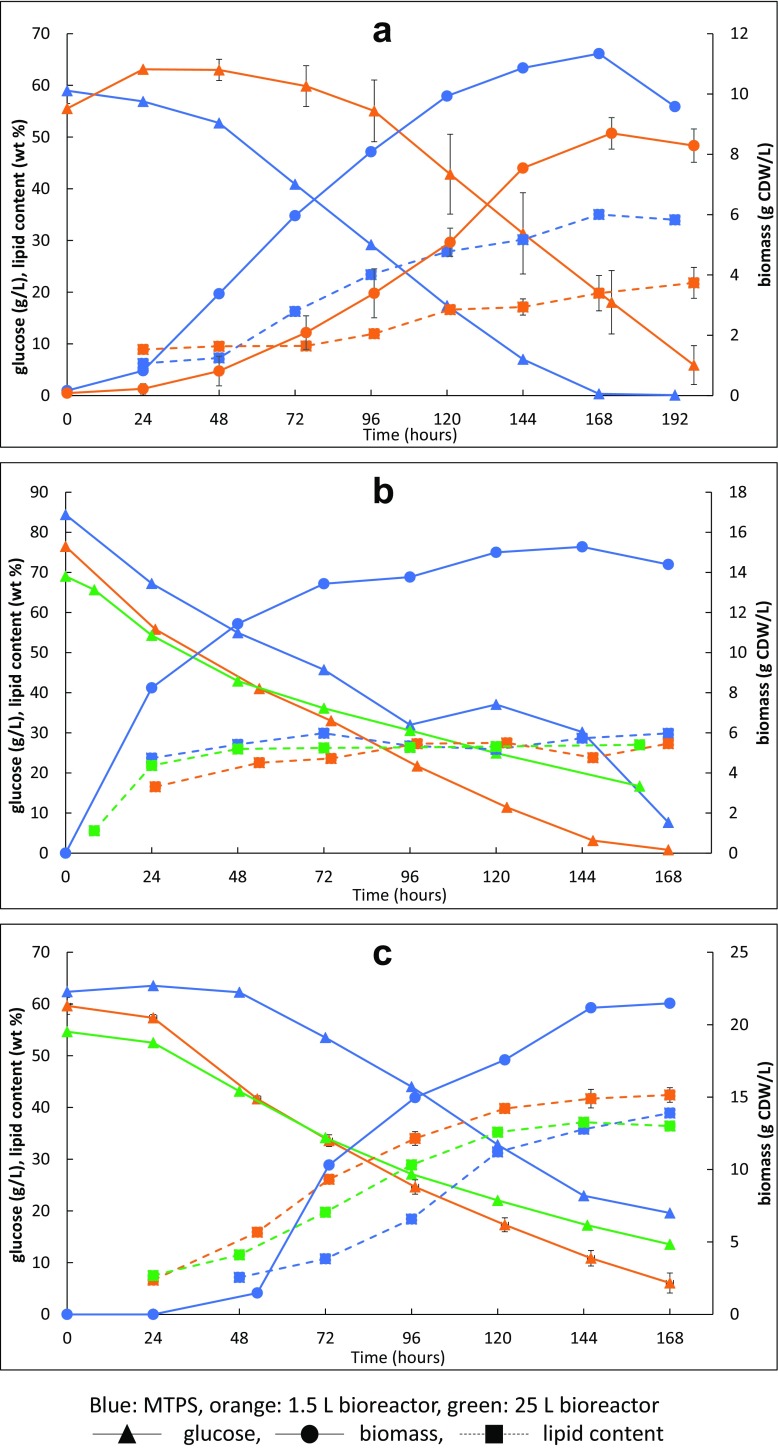
Fermentation characteristics of *C. cohnii* (**a**), *M. circinelloides* (**b**), and *M. alpina* (**c**) in Duetz-MTPS, 1.5 and 25 L bioreactors

Maximal glucose consumption rate was highest in the 1.5 L scale for the filamentous fungi (0.86 and 0.54 g/L/h for *M. circinelloides* and *M. alpina*, respectively) (Fig. 2). Maximal glucose consumption rate was the same in the MTPS and the 25 L bioreactor for *M. circinelloides* (0.72 g/L/h), while in case of *M. alpina*, it was higher in MTPS than in the 25 L bioreactor with 0.47—0.39 g/L/h. The dinoflagellate *C. cohnii* reached the same maximal glucose consumption rate in the MTPS and 1.5 L bioreactor (0.50 g/L/h). Comparison of biomass production rates between MTPS and bioreactors was only possible for *C. cohnii* due to significant wall growth of filamentous fungi *M. circinelloides* and *M. alpina* at all tested scales (Figure S5). Therefore, only the end-point biomass concentration of the fungi was measured from the bioreactor cultures. *C. cohnii* had the same maximal biomass production rate (0.11 g/L/h) in the MTPS and in the 1.5 L fermenter. The CO_2_ off-gas data from the bioreactor cultivations (show that after an exponential growth phase (10 h for *M. circinelloides* and 30 h for *M. alpina*), the cells entered into the stationary growth (i.e., lipid accumulation) phase (Figure S6). This is caused by the nitrogen depletion (approximately 1.5 and 5 g/L) from yeast extract. It is also visible from the growth and substrate consumption curves that *M. alpina* had 1 day longer lag phase in the MTPS than in the stirred bioreactors.

## Biomass concentration and lipid content of biomass

**Fig. 3:**
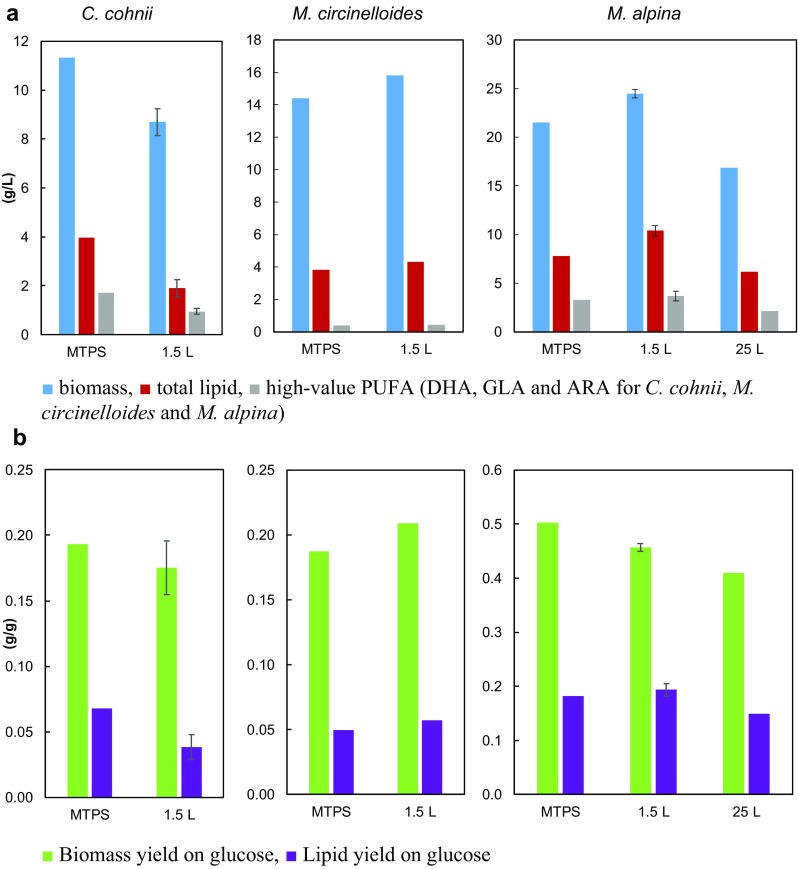
Comparison of physiological fermentation parameters of *C. cohnii*, *M. circinelloides* and *M. alpina* in Duetz-MTPS, 1.5 L bioreactor and 25 L bioreactor. (**a**) Biomass, total lipid, total high-value PUFA (DHA, GLA and ARA for *C. cohnii*, *M. circinelloides* and *M. alpina*) [g/L]. (**b**) Biomass yield on glucose, lipid yield on glucose [g/g]

Due to the long lag phase observed in the 1.5 L bioreactor runs with *C. cohnii*, the maximal biomass concentration was higher in the MTPS than in the glass bioreactor: 11.3 vs. 8.7 g/L (Fig. 3a). *M. circinelloides* reached comparable (14.4 and 15.8 g/L) end-point biomass concentrations in the MTPS and in the 1.5 L bioreactor (it was not measured at 25 L fermentation). The end-point biomass concentration of *M. alpina* in MTPS was higher than that in the 25 L bioreactor, but lower than that in the 1.5 L bioreactor: 21.5–24.5 ± 0 .5–16.9 g/L. Lipid content of *C. cohnii* increased during cultivations until glucose depletion, and reached significantly higher level in MTPS than in the glass bioreactor: 35.0 vs. 21.8 ± 3.0%. Oil content of *M. circinelloides* was above 20% already within the first 24–48 h in all tested scales (nitrogen source depleted at 10 h), and then, it increased only moderately in the following days with maximal values of 29.9–27.3—27.0% in MTPS, 1.5, and 25 L bioreactor runs, respectively. *M. alpina* started to accumulate lipids later than *M. circinelloides* due to the longer growth phase and higher nitrogen level in the medium (initial YE level was 10 g/L instead of 3 g/L). Maximal lipid content values of *M. alpina* were comparable across the tested scales: 36.4–42.4 ± 0.4–36.4% in MTPS, 1.5, and 25 L bioreactors.

## Fatty acid composition of single cell oil

The fatty acid composition of *C. cohnii*, *M. circinelloides*, and *M. alpina* biomass is summarized in the PCA scores plot of the GC data (Figure S7). *C. cohnii* is characterized by high content of C12:0, C14:0, C16:0 and C22:6n3 (DHA) and separated from the fungal oils on PC1 axis, while PC2 separates *M. circinelloides* from *M. alpina*. The separation of fungal oil composition is based on the presence/absence of C20 fatty acids (C20:3n6 DGLA and C20:4 ARA) and the relative amount of monounsaturated fatty acids (C16:1n7, C18:1n9c) in the oil. Maximum DHA content of the oil in *C. cohnii* microalga cells was 52.6 ± 4.3% in the bioreactor and 46.3% in MTPS (it increased from 42.9% after glucose depletion) (Table 1). It has been shown that *C. cohnii* has a high oxygen demand for growth and synthesis of highly unsaturated PUFA such as DHA (Hillig 2014). This is in agreement with our results since the oil of the microalga grown in the bioreactor—where the aeration is more effective than in Duetz-MTPS—contained more unsaturated fatty acids, (C18:1n9, C22:6n3 DHA) and less C14:0. In the case of *M. circinelloides*, the FA composition of the fungal oil matched very good in the MTPS and the 1.5 L bioreactor, while in 25 L bioreactor, the GLA content of oil was found to be higher (15.0% vs. 10.0%) (Table 2). Lipid content and fatty acid composition of the wall-grown fungi from the 1.5 L bioreactors was investigated, and it was found to be very similar to the submerged biomass (Table S2). The ARA content in *M. alpina* oil (42.0–35.3 ± 3.0–34.4%) showed a good match at different scales (Table 3); however, major differences were found in the oleic acid (C18:1n9) content between scales (11.7–24.8 ± 3.7–17.3%). Similar to *M. circinelloides*, the lipid content and composition of the wall-grown *M. alpina* biomass was very similar to the submerged biomass (Table S3).

**Table 1:** Fatty acid composition (%), lipid content of biomass (wt%) of *C.cohnii* ATCC 40750 in Duetz-MTPS and in 1.5 L benchtop bioreactors at 168 h (MTPS)–198 h (bioreactor)

	C12:0	C14:0	C16:0	C18:0	C18:1n9	C22:6n3	Lipid content (wt%)
2.5 mL	3.3	21.0	22.5	2.8	5.9	42.9	35.0
1.5 L	1.5 ± 0.5	11.6 ± 3.0	19.7 ± 1.3	2.5 ± 0.9	9.6 ± 1.0	52.6 ± 4.3	21.8 ± 3.0

**Table 2:** Fatty acid composition (%), lipid content of biomass (wt%) of *M. circinelloides* VI 04473 in Duetz-MPTS, 1.5 L benchtop bioreactor, and in 25 L pre-pilot bioreactor at 160–168 h

	C14:0	C16:0	C16:1	C18:0	C18:1n9	C18:2n6	C18:3n6	Lipid content (wt%)
2.5 mL	3.2	15.7	4.2	8.8	35.6	13.5	10.0	26.4
1.5 L	2.3	15.2	5.2	4.6	40.7	15.3	10.3	27.3
25 L	1.6	16.3	3.1	4.4	36.7	17.6	15.0	27.0

**Table 3:** Fatty acid composition (%), lipid content of biomass (wt%) of *M. alpina* ATCC 32222 in Duetz-MTPS, 1.5 L benchtop bioreactors, and 25 L pre-pilot bioreactor at 168 h

	C16:0	C18:0	C18:1n9	C18:2n6	C18:3n6	C20:3n6	C20:4n6	Lipid content (wt%)
2.5 mL	12.3	11.1	11.7	8.2	4.6	4.1	42.0	36.4
1.5 L	11.0 ± 0.2	9.6 ± 0.1	24.8 ± 3.7	4.3 ± 1.2	3.3 ± 0.2	2.3 ± 0.4	35.3 ± 3.0	42.4 ± 1.4
25 L	13.1	11.7	17.3	7.8	4.7	3.3	34.4	36.4

## FTIR analysis of *C. cohnii*, *M. circinelloides*, and *M. alpina* biomass

**Fig. 4:**
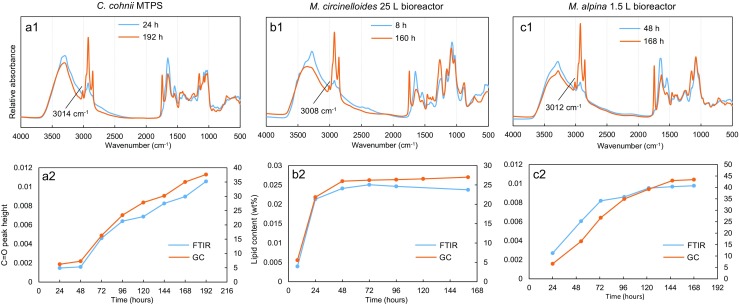
(a1–c1) FTIR spectra of *C. cohnii*, *M. circinelloides* and *M. alpina* at the first and last day of cultivation. (a2–c2) Lipid accumulation based on FTIR C=O ester peak height (from pre-processed spectra) and reference GC lipid content (wt%) data

Microalgal and fungal biomass were also analyzed by high-throughput FTIR spectroscopy. The most obvious change in mid-IR spectra of microalga and filamentous fungi during the bioprocesses was the increase of lipid-related peak intensities (Fig. 4). The C=O ester peak height at 1745 cm^−1^ in the mid-IR spectra was used to monitor lipid accumulation during cultivations, and these curves correlated well with reference curves for lipid content of biomass, obtained by the GC analyses. It is worth mentioning that the peak at approximately 3010 cm^−1^, which is related to =C–H stretching, correlates with the unsaturation level of single cell oil. The peak position was at 3014 cm^−1^ for *C. cohnii* (unsaturation index, UI = 2.86), 3012 cm^−1^ for *M. alpina* (UI = 1.93), and 3008 cm^−1^ for *M. circinelloides* (UI = 1.24). PCA results of FTIR data (Figure S8) confirm that biomass composition correlated well between different scales in case of *M. circinelloides* and *M. alpina*, while *C. cohnii* cultivation was less scalable from MTPS to 1.5 L bioreactors.

## Reproducibility in Duetz-MTPS

The reproducibility of *C. cohnii*, *M. circinelloides*, and *M. alpina* cultivations in MTPS were evaluated based on the fermentation results achieved in three individual wells of the same MTP (Table 4). *C. cohnii* and *M. circinelloides* showed good reproducibility after 8 and 7 days of cultivations with less than 15% coefficient of variation for all measured parameters (glucose consumption, fatty acid composition, pH, biomass concentration, and lipid content of the biomass), while in case of *M. alpina* the variations were higher.

**Table 4:** Well-to-well reproducibility of oleaginous microorganisms in 24-deepwell microtiter plates in the Duetz-system based on biomass production (cell dry weight, CDW, g/L), lipid content of the biomass (wt%), glucose consumption (g/L), and pH. Fermentation broths from three individual wells were analyzed from each MTP. *AVG* average, *CV%* coefficient of variation

	CDW (g/L)		Lipid content (wt%)		Glucose consumed (g/L)		pH	
	AVG	CV%	AVG	CV%	AVG	CV%	AVG	CV%
*C. cohnii* (*t* = 192 h)	9.5	4.1	34.0	7.9	58.9	0.03	6.9	2.3
*M. circinelloides* (*t* = 168 h)	13.3	2.3	29.5	13.6	76.1	1.3	5.0	1.7
*M. alpina* (*t* = 168 h)	20.6	8.0	36.5	17.7	40.2	20.6	6.6	0.2

## Discussion

*C. cohnii* had a much shorter lag phase in the MTPS than in the bioreactor (Fig. 2a) and it reached a substantially higher biomass concentration and lipid content than that in the stirred-tank bioreactor. Since the inoculation ratio was same at both scales (10 *v*/*v*%, OD_600nm_ = 4) this might be the consequence of high shear stress in the bioreactor, caused by the agitation on the cells (stirrer speed maximum was 530 rpm). Nonetheless, different inocula were used for the MTP and the bioreactor; therefore, no clear conclusion can be drawn. Hillig et al. cultivated *C. cohnii* in a 24-deepwell plate together with perfluorodecalin (PFD) in order to avoid (reduce) oxygen limitation. An OD (optical density) value of 17 with PFD compared to 13 without PFD was measured, while in our study, an OD of 31.6 was reached. Moreover, in the study by Hillig et al., addition of water to deepwell plates had to be applied during the long cultivation of *C. cohnii*, in order to compensate for the severe evaporation loss. In Duetz-MTPS, the evaporation rate is very low (16 μl/well/day at 30 °C, 50% humidity) (Enzyscreen); therefore, addition of water was not necessary in our study. The values achieved in the Duetz-MTPS with *C. cohnii* are promising in comparison with industrial requirements (CDW > 10 g/L, DHA in oil > 20%, total DHA > 1.5 g/L) (Kyle et al. 1998).

Oxygen transfer rate (OTR) was about 30 mmol O_2_ L/h with the applied settings in the Duetz-MTPS (Enzyscreen), which corresponds to a mass transfer coefficient (k_L_a) of approximately 150 1/h. This value is within the range (k_L_a of 100–300 1/h) that is typical for aerated, stirred-tank bioreactors (Duetz et al. 2000). Comparing the biomass, lipid content of biomass and fatty acid composition of the oil achieved in Duetz-MTPS and in bioreactors, it can be assumed that oxygen limitation was not an issue during microtiter plate cultivations of *C. cohnii*, *M. circinelloides*, and *M. alpina* oleaginous microorganisms.

For a better comparison of fermentation kinetics (Fig. 2 and Table S4) with filamentous fungi at different scales, a unified inoculation approach should have been applied (i.e., inoculation with spores and same final spores concentration).

The reproducibility results in the Duetz-MTPS can be explained by the difference in morphology between strains. Cell and spore suspension inocula were homogenous and easy to pipette in the case of *C. cohnii* and *M. circinelloides*, while the *M. alpina* inoculum in addition to spores also contained mycelium that made it difficult to transfer inoculum equally into each well. It is likely that separation (filtration) of mycelium fragments from spores or fragmentation of mycelium for *M. alpina* inoculation can decrease the observed variability in Duetz-MTPS cultivation (Knudsen 2015; Sohoni et al. 2012). The growth morphology of *M. alpina* is in the form of fluffy pellets of different sizes, and this can also negatively affect the reproducibility in the Duetz-MTPS. In order to reduce this effect, glass beads can be added to the cultivation. For example, Sohoni et al. observed for *Streptomyces coelicor* that the addition of 3 mm glass beads prevented both pellet morphology and wall growth, improving reproducibility and scalability from MTP to benchtop bioreactor (Sohoni et al. 2012). Another strategy to induce dispersed growth of filamentous fungi is the addition of carboxypolymethylene, an anionic polymeric additive to the medium (Knudsen 2015). Despite these issues, the fatty acid composition of all the tested microorganisms showed excellent reproducibility in the Duetz-MTPS (Table S1-S3).

In conclusion, key fermentation physiological parameters (glucose consumption rate, biomass concentration, lipid content of the biomass, biomass, and lipid yield) were comparable (max 30% difference) for the oleaginous fungi *M. circinelloides* and *M. alpina* in the Duetz-MTPS and benchtop or pre-pilot stirred-tank bioreactors (600–10,000× volumetric scale factors). This has been achieved despite the absence of control options, such as pH and DO, in the Duetz-MTPS, and the difficult fungal growth characteristics, such as severe wall growth. However, the heterotrophic microalga *C. cohnii* reached a significantly higher biomass and lipid concentration in the MTPS than in the 1.5 L bioreactor, probably due to shear force sensitivity of this species. It is worth mentioning that the screening throughput of oleaginous microorganisms in the Duetz-MTPS can be increased by combining at-line FTIR spectroscopy and the automation of the cultivation-analytical system (Li et al. 2016). Reproducibility and scalability results demonstrated that the Duetz-MTPS can be used for the cost-efficient, high-throughput screening of both single-cell and multicellular oleaginous microorganisms.

## Electronic supplementary material

ESM 1 (PDF 1.02 mb)
