# Supplementary material for: Assessment of the scalability of a microtiter plate system for screening of oleaginous microorganisms
Source: Appl Microbiol Biotechnol. 2018 Apr 11;102(11):4915–25. doi: 10.1007/s00253-018-8920-x (PMC5954000; doi:10.1007/s00253-018-8920-x)
Supplement: Supplementary file 1 — (PDF 1.02 mb) [file 253_2018_8920_MOESM1_ESM.pdf]

Applied Microbiology and Biotechnology

Assessment of the scalability of a microtiter plate system for screening of oleaginous microorganisms

Supplementary material

Gergely Kosa[1,*], Kiira S. Vuoristo[2], Svein Jarle Horn[2], Boris Zimmermann[1], Nils Kristian Afseth[3], Achim Kohler[1], Volha Shapaval[1]

(1) Faculty of Science and Technology, Norwegian University of Life Sciences, Postbox 5003, 1432 Ås, Norway

(2) Faculty of Chemistry, Biotechnology and Food Science, Norwegian University of Life Sciences, Postbox 5003, 1432 Ås, Norway

(3) Nofima AS, Osloveien 1, NO-1433 Ås, Norway

Phone number and e-mail address of the corresponding author: +47-454-46857, gergely.kosa@nmbu.no

Table of contents:

Table of contents:	Page
Micrographs of *C. cohnii* ATCC 40750	S3
Micrograph of single cell form *M. circinelloides* VI 04473	S3
Optical density vs. cell dry weight calibration of *C. cohnii* ATCC 4750	S4
pH in MTPs	S4
Wall growth of *M. alpina* ATCC 32222 in 42 L total volume bioreactor	S5
CO_2 off-gas data of bioreactor cultivations	S5
Fatty acid composition PCA	S6
FTIR spectra PCA	S7
Fatty acid composition of *C. cohnii* ATCC 4750	S8
Fatty acid composition of *M. circinelloides* VI 04473	S9
Fatty acid composition of *M. alpina* ATCC 32222	S10-11
Fermentation results	S11

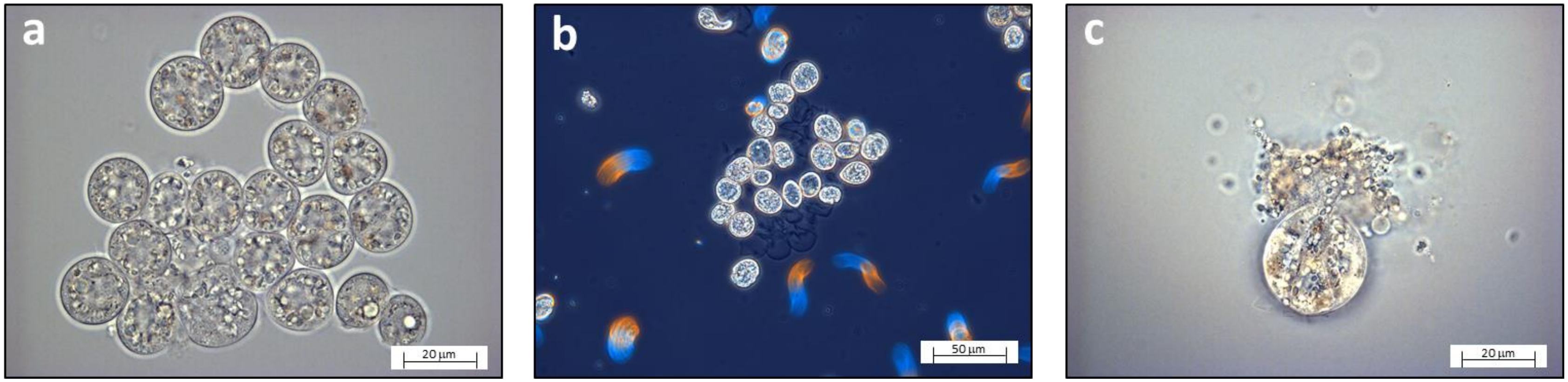

Fig. S1 Micrographs of *C. cohnii* ATCC 40750 in 1.5 L working volume benchtop bioreactor: **a)** cells contain high number of oval starch granules (48 h), **b)** phase-contrast image of moving cells and cysts (121 h), and **c)** bursting cell (76 h)

Fig. S2 Yeast-like form of the dimorphic fungus *Mucor circinelloides* (1.5 L working volume benchtop bioreactor, t = 12 h)

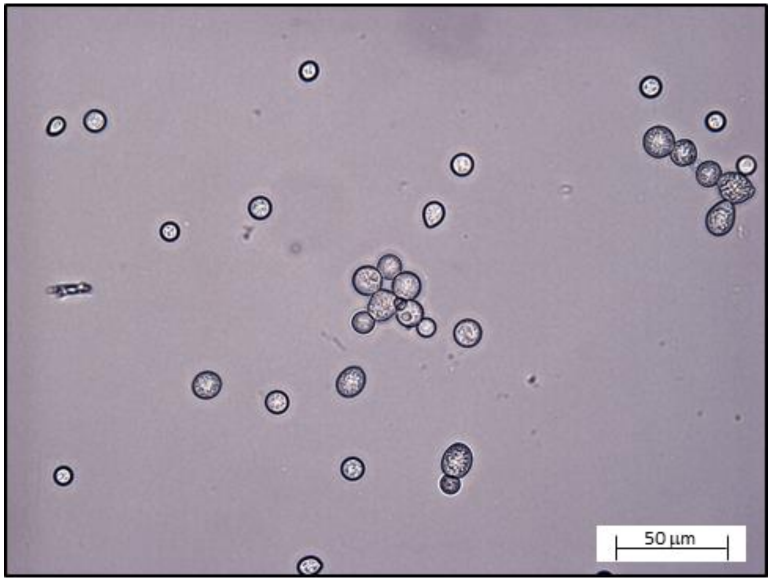

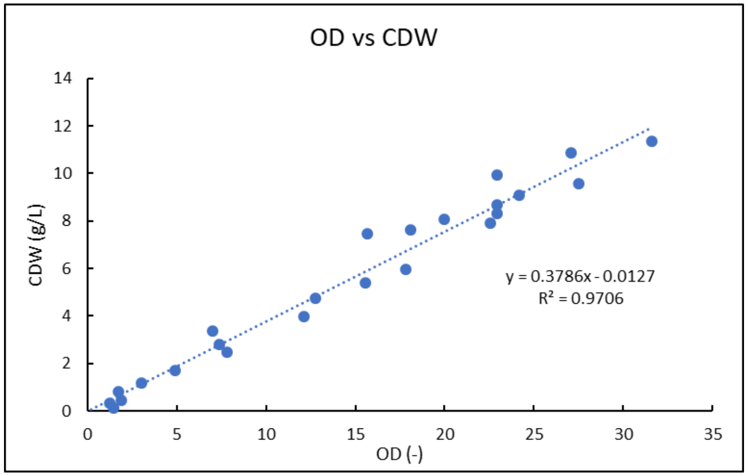

Fig. S3 Optical density (600 nm) and cell dry weight (CDW) calibration for *C. cohnii* ATCC 40750 (Data combined from Duetz-MTPS and 1.5 L working volume bioreactor cultivations)

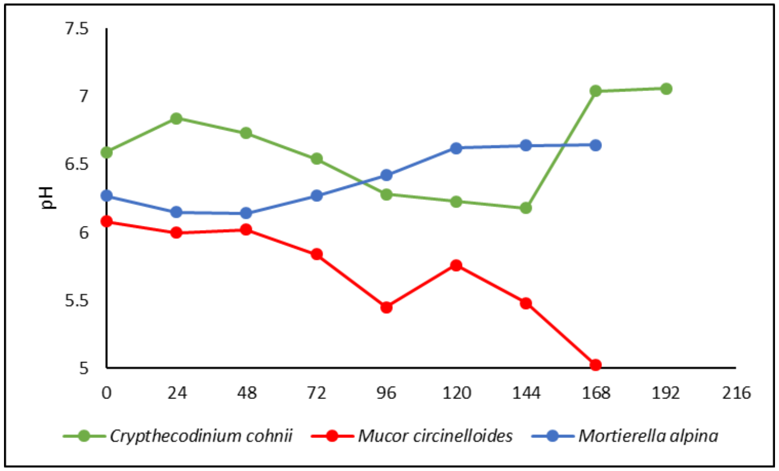

Fig. S4 Development of broth pH in Duetz-MTPS of *C. cohnii*, *M. circinelloides* and *M. alpina*. Media were not buffered.

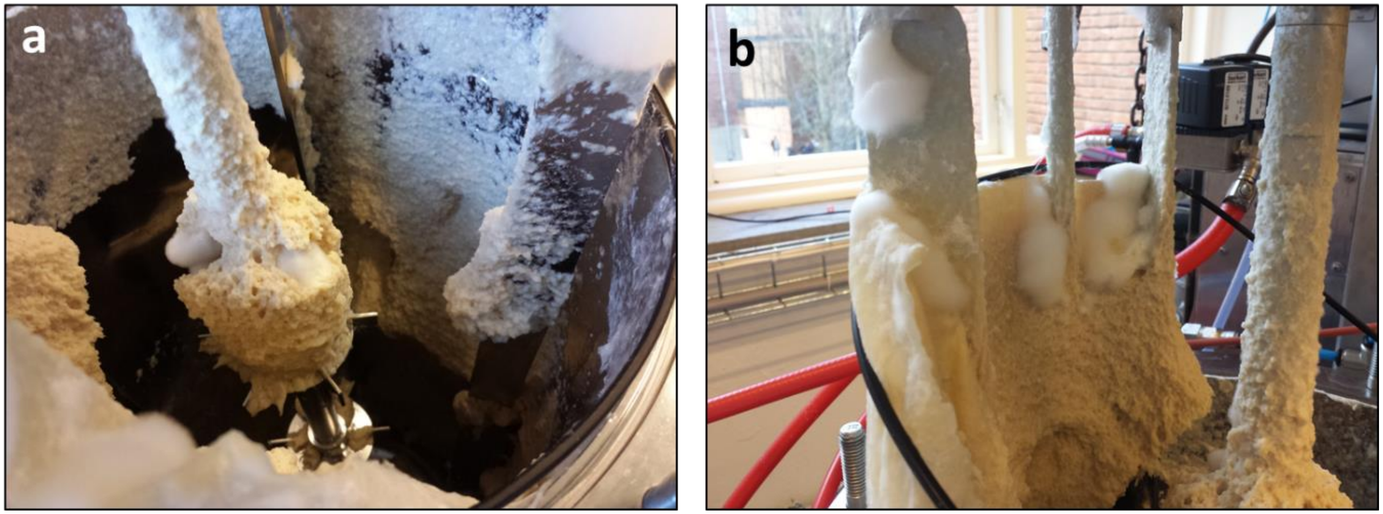

Fig. S5 *Mortierella alpina* ATCC 32222 grown on impellers, baffles and wall of the 42 L total volume pre.pilot bioreactor. Aerial mycelium started to grow on baffles.

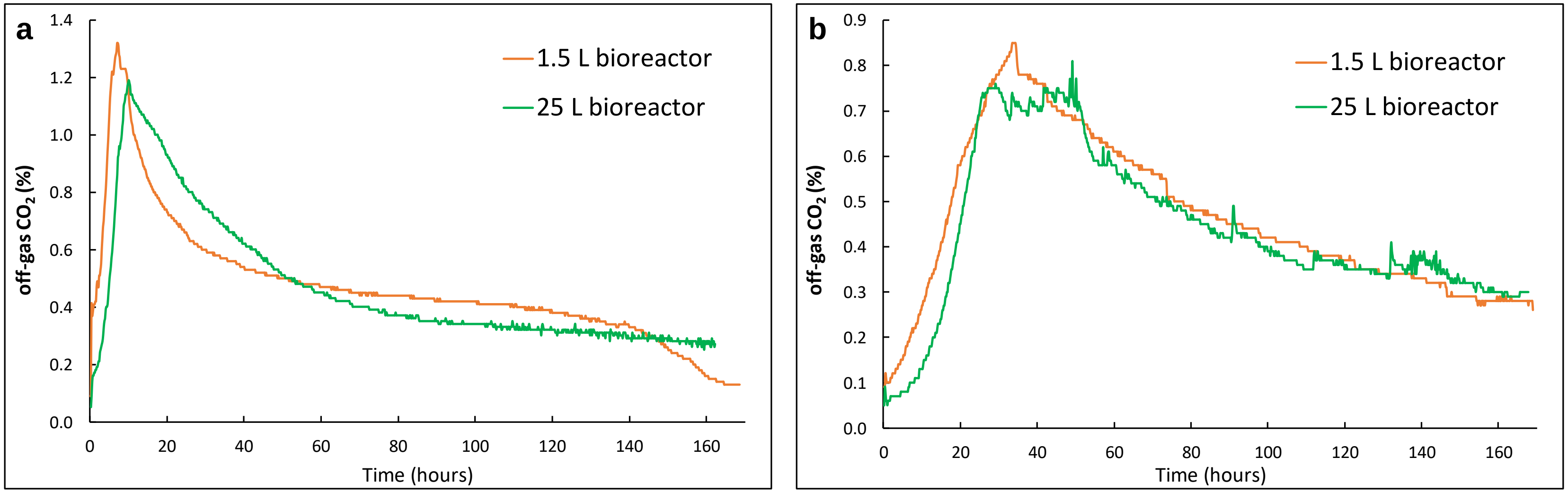

Fig. S6 CO_2 (%) in the exhaust gas during bioreactor cutlivations **a)** *M. circinelloides*, **b)** *M. alpina*

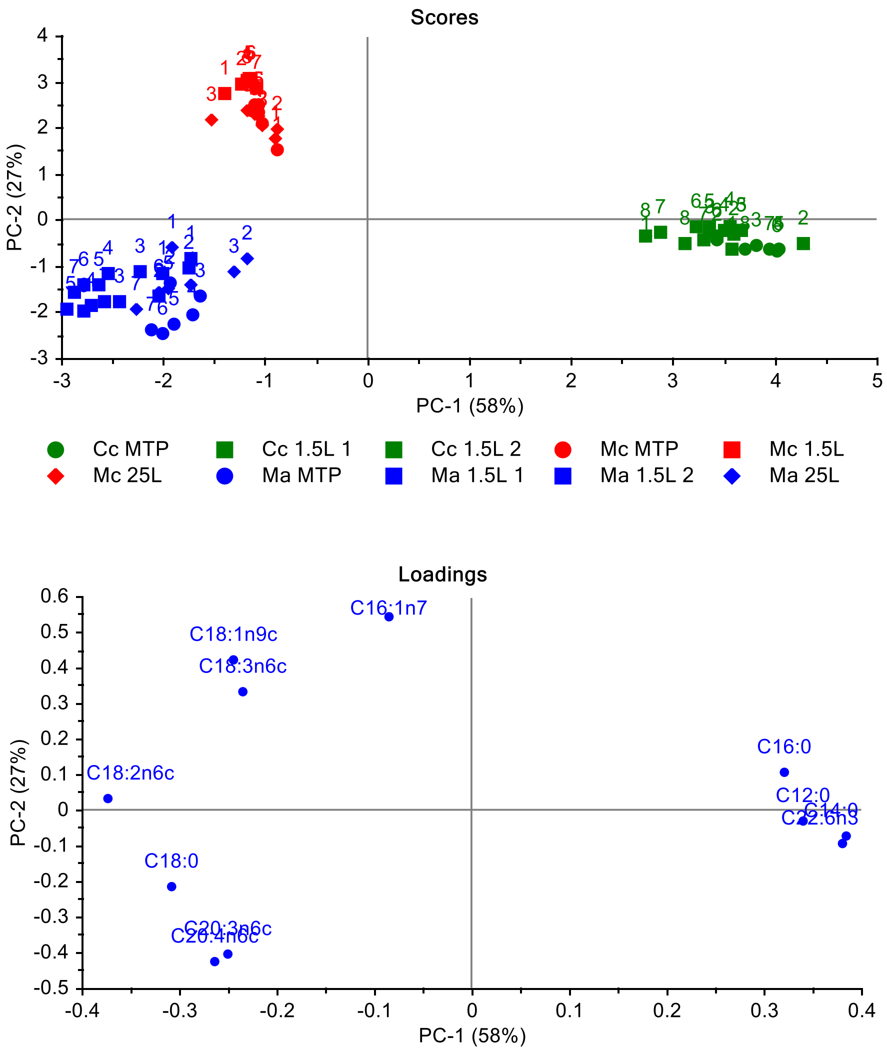

Fig. S7 a) PCA scores (samples) and **b)** loadings (fatty acids) of the fatty acid composition from *C. cohnii*, *M. circinelloides* and *M. alpina* cultured in Duetz-MTPS, 1.5 L benchtop and 25 L pre-pilot bioreactors. Numbers in the score plot indicate fermentation time in days.

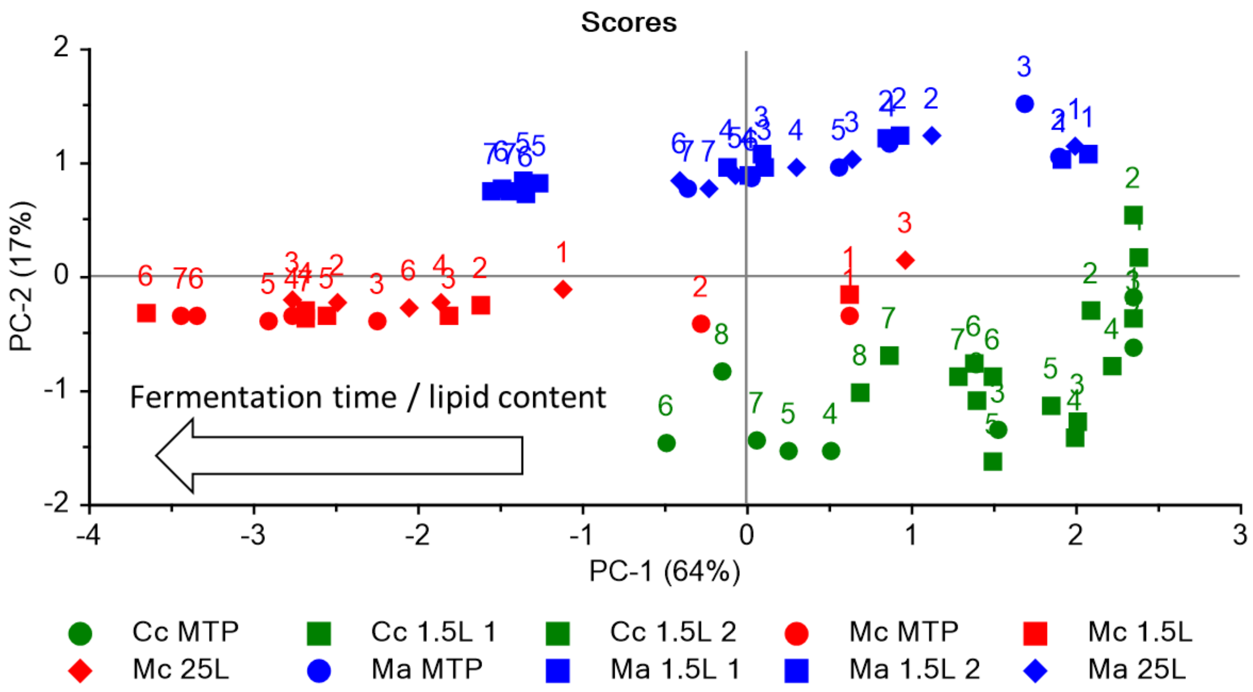

Fig. S8 PCA score plot of the (pre-processed) FTIR spectra of biomass (Cc = *C. cohnii*, Mc = *M. circinelloides*, Ma = *M. alpina*) at different cultivation scales. Numbers in the score plot indicate cultivation time in days.

Table S1 Fatty acid composition and lipid content (FAME, weight% of biomass) of *C. cohnii* ATCC 40750 in Duetz-MTPS, 1.5 L working volume benchtop bioreactor. Fatty acid composition results are average of three technical replicate extraction – GC measurements (if there was enough biomass). From Duetz-MTPS, three individual wells were also measured at 192 h for reproducibility test.

scale	time (h)	C12:0	C14:0	C16:0	C16:1n7c	C18:0	C18:1n9c	C20:0	C22:6n3	unsat. index	lipid content (wt%)
MTPS	24	0.7	10.8	17.8	0.4	1.4	10.3	0.0	58.3	3.61	6.2 ± 0.5
	48	1.6	14.7	21.5	0.2	1.2	7.3	0.0	51.2	3.19	7.3 ± 0.1
	72	3.0	19.4	22.0	0.2	2.1	6.8	0.1	44.6	2.78	16.3 ± 0.5
	96	3.7	21.3	22.2	0.1	2.3	6.1	0.2	42.6	2.64	23.4 ± 1.4
	120	3.7	21.5	22.5	0.1	2.7	5.9	0.2	42.0	2.60	27.8 ± 0.9
	144	3.7	21.4	22.0	0.1	2.6	5.7	0.2	43.1	2.66	30.2 ± 0.9
	168	3.3	21.0	22.5	0.1	2.8	5.9	0.2	42.9	2.65	35.0 ± 1.1
	192	2.6	18.9	21.8	0.1	2.6	6.0	0.2	46.3	2.86	34.0 ± 2.7
	192 well I	2.9	19.9	21.8	0.1	2.4	6.0	0.2	45.4	2.88	30.9
	192 well II	2.5	19.0	21.8	0.1	2.6	5.9	0.2	46.7	2.89	35.9
	192 well III	2.4	18.7	21.8	0.1	2.6	6.0	0.2	46.8	2.86	35.2
1.5 L WV bioreactor I	48	6.6	14.6	15.1	0.9	1.4	12.6	0.0	47.3	2.97	10.0
	76	3.8	14.5	17.9	0.8	1.4	10.9	0.0	49.0	3.08	9.1 ± 0.1
	97	3.5	14.8	19.6	0.7	1.0	10.4	0.0	48.6	3.04	11.4 ± 0.1
	121	3.6	15.4	21.3	0.5	1.0	10.7	0.1	45.8	2.87	16.3 ± 0.2
	144	2.9	14.5	20.6	0.4	1.7	9.8	0.2	47.9	3.01	17.1 ± 0.0
	172	2.3	14.8	21.4	0.3	2.8	9.4	0.2	47.2	2.95	22.2 ± 0.4
	198	1.9	13.7	20.6	0.2	3.1	8.8	0.3	49.5	3.29	23.9 ± 0.3
1.5 L WV bioreactor II	24	5.2	15.4	16.1	0.0	1.5	10.7	0.0	50.1	3.11	8.9
	48	8.1	16.3	17.7	0.6	1.3	8.7	0.0	45.3	2.82	9.1
	76	4.0	15.1	20.5	0.8	1.2	10.1	0.0	47.1	2.95	10.1 ± 0.0
	97	3.0	14.5	21.8	0.8	1.0	10.9	0.0	46.9	2.94	12.6 ± 0.2
	121	2.3	13.5	21.6	0.7	1.1	10.9	0.1	48.5	3.04	16.9 ± 0.1
	144	1.9	13.1	22.4	0.6	1.8	11.4	0.2	46.0	2.93	14.9 ± 0.5
	172	1.2	11.0	20.0	0.5	1.9	10.6	0.2	52.6	3.30	17.4 ± 0.1
	198	1.1	9.4	18.8	0.4	1.9	10.3	0.2	55.6	3.48	19.7 ± 0.0

Table S2 Fatty acid composition and lipid content (FAME, weight% of biomass) of *Mucor circinelloides* VI 04473 in Duetz-MTPS, 1.5 L working volume benchtop bioreactor and 25 L working volume pre-pilot bioreactor. Fatty acid composition results are average of three technical replicate extraction – GC measurements (if there was enough biomass). From Duetz-MTPS three individual wells were also measured at 168 h for reproducibility test.

scale	time (h)	C10:0	C12:0	C14:0	C15:0	C16:0	C16:1n5	C16:1n7	C17:0	C17:1	C18:0	C18:1n9t	C18:1n9c	C18:1n7c	C18:2n9t	C18:2n6c	C18:3n6c	C20:0	C20:1n9c	C20:2	C22:0	C24:0	unsat. index	lipid content (wt%)
MTPS	24	0.1	0.2	1.2	0.6	19.7	0.1	1.2	3.7	1.0	7.6	0.1	30.0	0.3	0.3	14.8	16.5	0.3	0.3	0.2	0.3	0.6	1.15	23.7 ± 1.3
	48	0.2	0.3	1.5	0.5	17.7	0.2	2.7	2.5	0.9	6.0	0.1	34.0	0.5	0.4	15.7	15.1	0.2	0.3	0.2	0.2	0.3	1.17	27.1 ± 0.4
	72	0.2	0.3	1.7	0.4	16.3	0.3	3.5	2.1	0.8	5.1	0.1	35.9	0.7	0.4	16.3	14.3	0.2	0.3	0.2	0.1	0.3	1.19	29.9 ± 0.4
	96	0.5	0.5	2.0	0.4	16.1	0.3	3.9	1.7	0.7	5.1	0.1	36.5	0.7	0.5	16.0	13.5	0.2	0.3	0.2	0.1	0.2	1.17	26.7 ± 0.6
	120	0.2	0.3	1.9	0.3	16.6	0.4	3.7	1.5	0.6	4.6	0.1	37.4	0.7	0.6	16.1	13.7	0.1	0.2	0.2	0.1	0.2	1.19	25.9 ± 1.1
	144	0.4	0.5	2.1	0.3	16.2	0.4	3.9	1.4	0.6	5.0	0.1	37.9	0.8	0.6	15.6	12.9	0.1	0.3	0.2	0.1	0.2	1.16	28.7 ± 1.1
	168	1.2	1.2	2.8	0.3	15.2	0.3	4.5	1.2	0.7	7.5	0.1	37.1	1.0	0.7	14.2	10.4	0.2	0.2	0.1	0.2	0.4	1.06	29.9 ± 3.4
	168 well I	1.0	1.2	2.8	0.3	14.9	0.3	4.6	1.2	0.7	7.3	0.1	37.6	1.1	0.7	14.4	10.4	0.2	0.2	0.1	0.1	0.3	1.06	31.0
	168 well II	0.9	1.1	2.6	0.3	15.0	0.3	4.6	1.3	0.7	6.9	0.1	37.9	1.0	0.7	14.5	10.6	0.2	0.3	0.1	0.2	0.3	1.08	34.0
	168 well III	1.2	1.1	2.7	0.3	15.2	0.3	4.5	1.2	0.6	7.0	0.1	37.4	1.0	0.7	14.4	10.8	0.2	0.2	0.1	0.2	0.3	1.07	28.0
1.5 L WV bioreactor	25	0.3	0.4	1.6	0.4	13.7	0.1	4.7	1.7	1.1	4.5	0.1	36.5	1.2	0.6	17.2	14.0	0.2	0.3	0.2	0.1	0.3	1.23	16.5 ± 0.9
	54	0.3	0.4	1.8	0.4	14.9	0.1	5.4	1.3	0.8	3.8	0.1	38.2	1.2	0.6	17.7	11.5	0.1	0.3	0.2	0.1	0.2	1.19	22.5 ± 1.2
	74	0.3	0.4	1.9	0.3	15.0	0.2	5.4	1.2	0.7	3.5	0.1	39.1	1.1	0.7	17.4	11.3	0.1	0.3	0.2	0.1	0.1	1.18	23.6 ± 0.5
	98	0.2	0.3	1.9	0.3	15.1	0.3	5.3	1.1	0.7	3.3	0.1	40.4	1.1	0.7	16.9	11.0	0.1	0.3	0.2	0.1	0.1	1.18	27.3 ± 2.5
	123	0.2	0.3	1.9	0.3	15.1	0.3	5.3	1.0	0.6	3.2	0.1	41.3	1.1	0.8	16.5	10.9	0.1	0.3	0.2	0.0	0.1	1.17	27.5 ± 1.4
	144	0.3	0.4	2.0	0.2	15.0	0.3	5.3	0.9	0.6	3.2	0.1	41.6	1.1	0.9	16.2	10.8	0.1	0.3	0.2	0.0	0.1	1.17	23.8 ± 1.5
	168	0.5	0.7	2.3	0.2	15.2	0.3	5.2	0.9	0.6	4.6	0.1	40.7	1.1	0.9	15.3	10.3	0.1	0.3	0.2	0.0	0.1	1.13	27.3 ± 1.0
	168 wall	0.6	0.8	2.4	0.2	15.1	0.2	5.2	0.9	0.6	6.1	0.1	39.6	1.2	0.9	14.8	9.9	0.2	0.3	0.2	0.1	0.2	1.09	29.5 ± 1.3
25 L WV bioreactor	8	0.0	0.3	1.6	0.6	15.3	0.0	2.0	1.2	0.4	6.0	0.0	21.6	0.4	1.6	15.7	31.2	0.0	0.0	0.2	0.4	1.3	1.53	4.51 ± 0.1
	24	0.1	0.2	1.3	0.6	19.7	0.1	2.4	2.9	0.9	7.5	0.1	31.0	0.5	0.4	17.0	13.0	0.3	0.3	0.2	0.2	0.5	1.10	21.8
	48	0.1	0.2	1.5	0.5	19.6	0.1	2.8	2.2	0.8	6.6	0.1	32.6	0.6	0.5	17.5	12.4	0.2	0.2	0.2	0.2	0.4	1.11	26.0
	72	0.1	0.2	1.5	0.4	18.4	0.2	2.8	1.8	0.7	5.9	0.1	33.3	0.6	0.4	18.2	13.7	0.2	0.2	0.2	0.2	0.3	1.17	26.2
	96	0.1	0.2	1.6	0.3	17.5	0.3	3.1	1.5	0.6	5.0	0.1	35.1	0.6	0.5	17.9	14.0	0.2	0.2	0.2	0.1	0.3	1.20	26.4
	120	0.1	0.2	1.6	0.3	17.0	0.4	3.0	1.4	0.6	4.7	0.1	35.6	0.6	0.5	17.8	14.6	0.1	0.2	0.2	0.1	0.3	1.22	26.6
	160	0.1	0.2	1.6	0.3	16.3	0.5	3.1	1.2	0.5	4.4	0.1	36.7	0.7	0.6	17.6	15.0	0.1	0.2	0.2	0.1	0.2	1.24	27.0

Table S3 Fatty acid composition and lipid content (FAME, weight% of biomass) of *Mortierella alpina* ATCC 32222 in Duetz-MTPS, 1.5 L working volume benchtop bioreactor and 25 L working volume pre-pilot bioreactor. Fatty acid composition results are average of three technical replicate extraction – GC measurements (if there was enough biomass). From Duetz-MTPS three individual wells were also measured at 168 h for reproducibility test.

scale	time (h)	C14:0	C15:0	C16:0	C16:1n7c	C17:0	C17:1	C18:0	C18:1n9c	C18:1n7c	C18:2n6c	C18:3n6c	C20:0	C20:1n9c	C20:2	C20:3n6c	C20:4n6c	C22:0	C24:0	unsat. index	lipid content (wt%)
MTPS	48	0.9	1.0	12.0	0.4	1.6	0.8	5.0	11.8	0.3	10.4	15.0	0.3	0.3	0.3	5.0	30.3	0.7	1.1	2.23	7.1 ± 0.2
	72	1.4	1.0	16.3	0.3	2.1	0.7	6.0	14.3	0.3	7.2	9.2	0.4	0.2	0.2	5.6	30.6	0.7	1.1	2.02	10.7 ± 0.3
	96	1.7	0.4	16.5	0.2	1.0	0.3	9.6	13.8	0.2	7.1	5.6	0.6	0.3	0.3	4.9	34.3	0.9	0.9	2.01	18.4 ± 1.1
	120	1.4	0.2	14.5	0.1	0.6	0.1	11.0	12.4	0.2	8.2	4.9	0.6	0.4	0.4	4.2	38.0	0.9	0.7	2.13	31.4 ± 1.1
	144	1.1	0.2	12.9	0.1	0.5	0.1	10.9	10.6	0.2	8.1	5.1	0.6	0.4	0.4	4.2	42.2	0.8	0.6	2.28	35.8 ± 1.6
	168	0.9	0.2	12.4	0.1	0.5	0.1	11.1	12.2	0.2	8.7	4.6	0.6	0.5	0.5	4.0	40.7	0.9	0.6	2.23	38.9 ± 2.2
	168 well I	1.0	0.2	12.7	0.1	0.5	0.1	11.3	12.7	0.2	8.9	4.5	0.6	0.5	0.5	4.0	39.4	0.9	0.5	2.23	40.4
	168 well II	0.9	0.2	12.3	0.1	0.5	0.1	11.0	12.2	0.2	9.0	4.5	0.6	0.5	0.5	3.8	40.7	0.9	0.6	2.11	40.0
	168 well III	1.5	0.0	15.9	0.2	0.4	0.1	10.1	14.1	0.2	6.0	5.1	0.6	0.5	0.3	4.9	37.9	0.9	0.6	2.27	29.0
1.5 L WV bioreactor I	24	0.7	2.7	9.1	0.3	4.9	2.9	1.5	12.9	0.6	8.3	16.2	0.0	0.3	0.3	4.4	29.3	0.3	1.2	2.22	6.6 ± 0.2
	53	1.5	0.9	16.8	0.2	2.2	0.9	7.6	21.5	0.3	5.8	5.7	0.5	0.4	0.3	3.7	26.6	0.9	1.2	1.76	16.4 ± 0.1
	73	1.2	0.4	15.1	0.2	1.3	0.4	9.6	23.7	0.3	5.3	4.1	0.7	0.7	0.3	3.0	29.2	1.1	1.1	1.79	26.7 ± 0.3
	97	0.9	0.3	13.0	0.1	0.9	0.3	10.5	24.6	0.2	4.9	3.6	0.7	0.9	0.3	2.7	31.4	1.2	1.1	1.86	34.9 ± 0.8
	122	0.7	0.2	12.1	0.1	0.8	0.2	10.4	26.2	0.2	4.3	3.4	0.7	1.1	0.3	2.4	31.8	1.2	1.1	1.88	39.2 ± 2.2
	146	0.5	0.2	11.5	0.1	0.6	0.2	10.2	23.3	0.2	5.0	3.4	0.7	1.0	0.3	2.6	35.2	1.2	1.0	2.00	43.0 ± 0.8
	168	0.5	0.1	10.9	0.1	0.6	0.1	9.6	22.2	0.2	5.2	3.4	0.7	1.0	0.4	2.6	37.4	1.2	1.1	2.08	43.4 ± 0.4
1.5 L WV bioreactor II	24	0.6	2.9	8.7	0.3	5.1	3.1	1.5	11.9	0.3	8.2	15.7	0.1	0.2	0.3	4.4	29.8	0.2	1.3	2.23	6.7 ± 0.1
	53	1.5	1.0	16.2	0.2	2.4	1.0	7.2	22.0	0.3	5.4	6.4	0.5	0.5	0.3	3.6	25.9	0.9	1.3	1.75	15.4 ± 0.0
	73	1.2	0.5	14.8	0.2	1.4	0.5	9.2	25.8	0.3	4.5	4.2	0.7	0.8	0.2	2.8	27.4	1.1	1.2	1.74	25.5 ± 0.4
	97	0.9	0.3	13.0	0.1	1.0	0.3	10.1	27.8	0.2	4.0	3.6	0.7	1.1	0.2	2.4	29.0	1.2	1.1	1.78	33.1 ± 0.3
	122	0.7	0.2	12.1	0.1	0.8	0.2	10.3	26.2	0.2	4.3	3.4	0.7	1.1	0.3	2.4	31.7	1.2	1.1	1.88	40.4 ± 2.1
	146	0.6	0.2	11.5	0.1	0.7	0.2	9.8	28.0	0.2	3.5	3.2	0.7	1.2	0.2	2.1	31.7	1.2	1.2	1.88	40.4 ± 0.0
	168	0.5	0.2	11.1	0.1	0.6	0.2	9.5	27.4	0.2	3.5	3.2	0.7	1.2	0.2	2.0	33.2	1.2	1.1	1.93	41.4 ± 0.3
25 L WV bioreactor	24	0.7	2.3	10.7	0.2	5.1	1.8	2.8	14.3	0.2	8.1	19.3	0.0	0.3	0.3	4.8	24.4	0.4	1.3	2.08	7.5 ± 0.9
	48	1.5	2.4	15.4	0.3	5.1	1.9	4.2	14.1	0.2	5.6	10.4	0.3	0.2	0.3	4.9	28.2	0.6	1.3	1.94	11.5 ± 0.3
	72	1.7	0.9	18.0	0.2	2.3	0.7	8.6	16.8	0.2	6.5	6.3	0.6	0.4	0.3	4.5	28.4	1.0	1.2	1.81	19.8 ± 0.2
	96	1.2	0.5	15.8	0.2	1.5	0.4	10.5	17.4	0.2	7.1	5.3	0.7	0.5	0.3	3.9	30.9	1.1	1.0	1.88	28.9 ± 0.3

	120	0.8	0.3	14.2	0.1	1.2	0.3	11.5	17.7	0.2	7.4	4.9	0.8	0.6	0.4	3.5	32.3	1.2	1.0	1.92	35.2 ± 1.0
	144	0.7	0.3	13.5	0.1	1.1	0.3	11.8	17.4	0.2	7.6	4.8	0.8	0.7	0.4	3.4	33.3	1.1	0.9	1.96	37.1 ± 2.4
	168	0.6	0.3	13.1	0.1	1.0	0.2	11.7	17.3	0.2	7.8	4.7	0.7	0.7	0.4	3.3	34.4	1.1	0.8	2.00	36.4 ± 0.7
	168 wall	0.8	0.6	13.2	0.1	1.7	0.5	10.3	16.3	0.2	6.9	5.5	0.7	0.6	0.4	3.7	34.1	1.2	1.1	2.00	35.2 ± 0.4

Table S4 Fermentation results of *C. cohnii* (168 – 192 h), *M. circinelloides* (168 h) and *M. alpina* (160-168 h) cultivated in Duetz-MTPS, 1.5 L and 25 L working volume bioreactors. Yx/s: biomass yield on glucose, Yp/s: lipid yield on glucose, Qp: overall lipid production rate, Qx: overall biomass production rate, Qs: overall substrate consumption rate, qP: overall specific lipid production rate, qS: overall specific substrate consumption rate.

C. cohnii	Biomass (g CDW/L)	Total lipid (g/L)	DHA (g/L)	Glu consumed (g/L)	Yx/s (g/g)	Yp/s (g/g)	Qp (g/L/day)	Qx (g/L/day)	Qs (g/L/day)	qP (g/g/day)	qS (g/g/day)
MTPS	11.3	4.0	1.70	58.6	0.193	0.068	0.57	1.62	8.4	0.050	0.739
1.5 L	8.3±0.6	1.8±0.4	0.9±0.1	49.6±2.8	0.167±0.021	0.036±0.009	0.219±0.045	1.005±0.067	6.0±0.3	0.026±0.004	0.726±0.09

M. circinelloides	Biomass (g CDW/L)	Total lipid (g/L)	GLA (g/L)	Glu consumed (g/L)	Yx/s (g/g)	Yp/s (g/g)	Qp (g/L/day)	Qx (g/L/day)	Qs (g/L/day)	qP (g/g/day)	qS (g/g/day)
MTPS	14.4	3.8	0.38	76.8	0.188	0.050	0.543	2.056	11.0	0.038	0.762
1.5 L	15.8	4.3	0.45	75.7	0.209	0.057	0.617	2.261	10.8	0.039	0.683
25 L	-	-	-	52.3	-	-	-	-	-	-	-

M. alpina	Biomass (g CDW/L)	Total lipid (g/L)	GLA (g/L)	ARA (g/L)	Glu consumed (g/L)	Yx/s (g/g)	Yp/s (g/g)	Qp (g/L/day)	Qx (g/L/day)	Qs (g/L/day)	qP (g/g/day)	qS (g/g/day)
MTPS	21.5	7.8	0.36	3.3	42.8	0.502	0.183	1.12	3.07	6.1	0.052	0.284
1.5 L	24.5±0.5	10.4±0.5	0.34±0.03	3.7±0.5	53.6±1.9	0.46±0.01	0.194±0.011	1.48±0.08	3.5±0.03	7.7±0.3	0.061±0.003	0.313±0.005
25 L	16.9	6.1	0.29	2.1	41.1	0.410	0.149	0.92	2.5	5.9	0.055	0.366
